# IoT-Based Research Equipment Sharing System for Remotely Controlled Two-Photon Laser Scanning Microscopy

**DOI:** 10.3390/s21041533

**Published:** 2021-02-23

**Authors:** Eunwoo Park, Jaehyun Lim, Byung Cheol Park, Daekeun Kim

**Affiliations:** 1 Advanced Photonics Research Institute, Gwangju Institute of Science and Technology, Gwangju 61005, Korea; statice13@gist.ac.kr; 2 Department of Mechanical Engineering, Dankook University, Yongin 16890, Korea; LOMM_jhlim@dankook.ac.kr; 3 Department of Dermatology, College of Medicine, Dankook University, Cheonan 31116, Korea; 4exodus@dankook.ac.kr

**Keywords:** IoT, remote control, remote operation, remote sharing economy, research equipment sharing, two-photon laser scanning microscopy, MQTT

## Abstract

In this study, two-photon laser scanning microscopy (TPLSM) based on the internet of things (IoT) is proposed as a remote research equipment sharing system, which enables the remote sharing economy. IoT modules, where data are transmitted to and received from the remote users in the web service via IoT, instead of a data acquisition (DAQ) system embedded in the conventional TPLSM, are installed in the IoT-based TPLSM (IoT-TPLSM). The performance for each IoT module is evaluated independently, and it is confirmed that it works well even in a personal computer-free environment. In addition, a message queuing telemetry transport (MQTT) protocol is applied to the DAQ interface in the web service, and a graphic user interface for enabling the remote users to operate IoT-TPLSM remotely is also designed and implemented. For the image acquisition demonstration, the stained cellular images and the autofluorescent tissue images are obtained in IoT-TPLSM. Lastly, it is confirmed that the comparable performance is provided with the conventional TPLSM by evaluating the imaging conditions and qualities of the three-dimensional image stacks processed in IoT-TPLSM.

## 1. Introduction

With the advent of the Fourth Industrial Revolution (4IR) worldwide, the integration of diverse networks between industries has formed a hyper-connected society. All industries converge with each other and develop together, as described in “Industry 4.0” [1,2]. For example, in manufacturing, a smart factory where a series of processes is linked to each other, unlike conventional automation only applied to an individual unit process, is aimed to optimize the operation by upgrading the production process and ensuring flexibility [3]. Accordingly, the national policies on the 4IR are presented, and its utilization methods in various fields are actively discussed. One of the representative 4IR applications is the sharing economy or the on-demand economy that connects social demand and supply, such as car-sharing or home-sharing. It has spread out through a digital platform to establish a new economic structure and maximize resource usage [4].

The concept of a sharing economy can be applied to research equipment, which is essential to do research but too expensive to own. Before the sharing economy arose, several trial systems to share research equipment had been developed. At the government level, a system for joint use of equipment, such as “e-Tube” or “ZEUS”, has been established in South Korea to utilize national research facilities and idle equipment [5]. Similarly, the UK operates an equipment sharing policy called “Equipment.data”, which promotes sharing of equipment between universities [6,7]. Both systems provide the database for basic information, current status, and services on the list of sharable equipment, but the user should ask the usage schedule to the equipment operator individually and visit onsite with a sample to use it [8]. At the company level, a German company provides an inventory software solution that tracks samples, specimens, consumables, and chemicals in the laboratory [9]. It was only for management purposes used to schedule the equipment or check its condition, and it is impossible to check the equipment conditions from outside since users must be in the same network to share content. At the university level, research equipment sharing services have been initiated in the Netherlands and the United States [10,11] but are operated closed, according to each university’s internal policy. Eventually, users should be onsite with a sample since these systems or services are mostly limited to provide information for sharable equipment only. In the joint equipment support facilities, users can utilize equipment, but they have the hassle of visiting the facility in person to utilize the equipment. Moreover, the use of equipment not provided by these facilities must be additionally approved for both the visit and the use by individually contacting the institution that owns it. One possible suggestion to resolve these problems is to send a sample to be processed or measured to the facility or the institution. However, the equipment operators have specialized knowledge for the equipment itself but are not familiar with users’ samples, resulting in compelling equipment users to still visit the facility or the institution with samples.

Currently, the concept of a remote sharing economy is proposed, which is similar to remote surgery. The only difference is that users located at a distance share equipment during the teleoperation, whereas surgeons operate on a patient located at a distance during the remote surgery. The user sends a sample to the institution that owns the necessary equipment, and the institution operator loads the sample into the equipment according to schedule. Then, the remote user can obtain the results by operating the equipment as desired. Equipment sharing based on remote operation gives several advantages over the conventional one. First, all the users can use sharable equipment provided by the facilities and the institutions worldwide once the sample is delivered and ready to use. Second, they do not have extra travel to conduct the experiment, resulting in saving time. Third, there are not any security issues from access by outsiders when they visit facilities or institutions.

There have been several studies on remote equipment operation over the Internet [12,13]. These studies used their equipment-specific protocols to operate equipment remotely and have a limitation to expand to other equipment. Recently, the Internet of Things (IoT) [14], one of the representative 4IR technologies, has been introduced and is extensively used for exchanging data between devices, mostly by monitoring signals from various sensors [15,16,17]; it also has been employed in controlling systems [18]. Nevertheless, IoT-based remote operation has not been applied to share equipment up to now, and demand on standard protocol still exists for sharing pieces of equipment.

In this paper, an IoT-based remote operation system for sharing equipment is proposed. As a piece of sharable equipment, two-photon laser scanning microscopy (TPLSM) is selected, which have IoT capability via a message queuing telemetry transport (MQTT), the standard protocol for IoT messaging between the equipment and its users. It is designed and built for a computer-independent system by combining independent IoT-based modules for the actuators and sensors. A web service for IoT-based TPLSM (IoT-TPLSM) is also implemented to manage and operate it remotely, including a database and MQTT broker. Its performance was evaluated from the image quality, and its potential for sharing equipment via standard protocols was confirmed by remotely acquiring 3D images for biological samples. The proposed remote sharing system gives remote users a high degree of freedom of operation in a stable network via IoT, providing a realistic solution for the sharing of equipment.

## 2. Materials and Methods

### 2.1. Hardware Design

**Figure 1:**
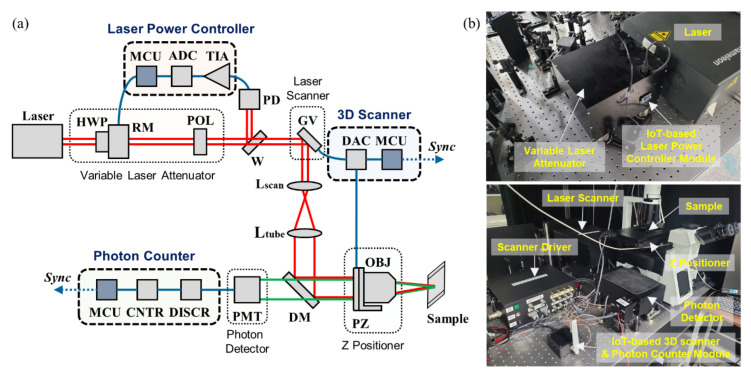
(**a**) Hardware configuration of the Internet of Things two-photon laser scanning microscopy (IoT-TPLSM). (**b**) Photograph of the customized modules. HWP: half-wave plate; RM: rotary motor; W: optical window; PD: photodiode; TIA: trans-impedance amplifier; GV: galvanometer mirror; L: optical lens; DM: dichroic mirror; PZ: piezo stage; OBJ: objective lens; PMT: photomultiplier tube; DISCR: discriminator; CNTR: pulse counter; MCU: microcontroller unit.

**Figure A1:**
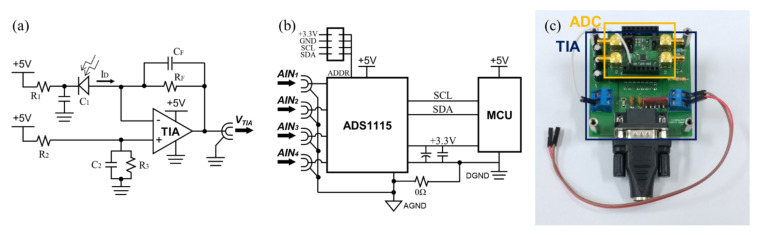
Electronic schematics for (**a**) the trans-impedance amplifier (TIA) circuit and (**b**) the analog-to-digital converter (ADC) circuit. (**c**) A photograph of the combined circuit boards for the laser power controller module.

**Figure A2:**
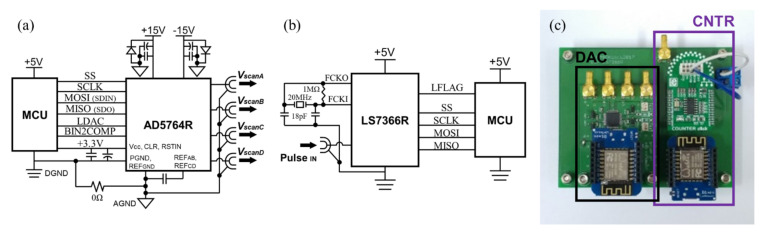
Electronic schematics for (**a**) the digital-to-analog converter (DAC) circuit and (**b**) the pulse counter (CNTR) circuit. (**c**) A photograph of the combined circuit boards for the 3D scanner module and the photon counter module.

The overall hardware configuration for IoT-TPLSM, a 3D fluorescence microscope with IoT capability, is shown in Figure 1. A customized TPLSM consists of four major components, which are the variable laser attenuator, galvanometer, Z positioner, and photon detector: the variable laser attenuator adjusts the laser power manually. The galvanometer and z positioner move the focal spot laterally and axially, respectively. Photon detector identifies optical pulse. Its users operate the TPLSM with their personal computer (PC) with the data acquisition (DAQ) system embedded in the PC and obtained 3D image stacks in the graphic user interface (GUI) implemented on the PC. On the other hand, IoT-TPLSM functions with microcontroller unit (MCU) development boards (WeMos D1 mini pro, WeMos Electronics) for WiFi MCU (ESP8266, Espressif, Shanghai, China) in a PC-free environment, instead of a DAQ system and GUI of a PC; that is, the existing DAQ system is replaced with these MCUs, and a GUI is substituted with web services via IoT. These MCUs are combined with an analog-to-digital converter (ADC), a digital-to-analog converter (DAC), or a counter (CNTR) circuit, as shown in Figure A1 and Figure A2. These IoT-based modules include a laser power controller, a 3D scanner, and a photon counter, as explained in Figure 1a.

#### 2.1.1. Laser Power Controller Module

A tunable Ti:Sapphire femtosecond pulsed laser (Chameleon Vision II, Coherent, Santa Clara, CA, USA) was used as the light source for TPLSM. It has a repetition rate of 80 MHz, and its average optical power was up to about 3.0 W at a wavelength of 800 nm without a power attenuator. Since photodamage may occur when excessive light is irradiated on the sample [19], a laser optical attenuator should be additionally required to adjust the proper light intensity on the sample. The customized variable laser attenuator unit was designed based on the light polarization. The laser output was horizontally polarized, and a half-wave plate (10RP02-46, Newport, Irvine, CA, USA) was manually rotated to change the light polarization direction, resulting in controlling the light intensity through a polarizer (GL10-B, Thorlabs, Newton, NJ, USA).

The laser power controller module was designed to regulate laser power automatically to the reference input power value given via IoT in a variable laser attenuator unit. It measured a certain percentage (about 0.19% at a wavelength of 800 nm) of the light reflected by a laser window (WG11010-B, Thorlabs) at a Si-photodiode (FDS10X10, Thorlabs) and actuated a rotary motor (T-RSW60C, Zaber Technologies, Vancouver, BC, Canada) where a half-wave plate was mounted. A closed-loop controller for laser power was implemented with MCU. The customized trans-impedance amplifier (TIA) circuit in photoconductive mode converted the photocurrent *I_D_* into the output voltage *V_TIA_* according to Equation (1):

(1) $$V_{TIA}=V_{CC}\cdot \left(\frac{R_{3}}{R_{2}+R_{3}}\right)-R_{F}\cdot I_{D}$$

where *V_TIA_* is a measured voltage indicating the light intensity, *V_CC_* is a supply voltage of a circuit, *I_D_* is a photocurrent generated by the photodiode, and *R* is resistance at each point in Figure A1a. The resistance values were selected so that the output voltage represented the light intensity used in the TPLSM. The voltage *V_TIA_* converted from the light intensity was transferred to the MCU through a 16-bit ADC (ADS1115, Texas Instruments), as seen in Figure A1b. The feedback loop in the laser power controller module was constructed so that the MCU made the measured light intensity reach the target one given by the remote users in the web service via IoT by controlling a rotary motor.

#### 2.1.2. 3D Scanner Module

In the conventional TPLSM, the lateral raster scan and the axial scan are cross-repeated to obtain a 3D volumetric image with the DAQ system in a PC. For the lateral raster scan, the galvanometer XY mirrors (6210H, Cambridge Technology, Bedford, MA, USA) in the laser scanner unit steer the focal spot into the lateral position. The maximum scanner driving voltage (*V_Scan_*) to cover the field of view (FOV) was determined according to Equation (2):

(2) $$V_{Scan}=\left\{\arctan\left(\frac{FOV}{2000}\cdot \frac{FL_{Tube}}{FL_{Obj}+FL_{Scan}}\right)\right\}\times \left\{\frac{180{}^{\circ}}{\pi }\cdot C_{Mirror}\cdot C_{Obj}\right\}.$$

The term enclosed in the first brace is for calculating the optical angle from an optical lens configuration. *FOV* is the FOV on the image plane in μm, and *FL* in mm is a focal length of the lens that corresponds to the subscript. The focal lengths of the scan lens, the tube lens, and the objective lens (UPLFLN 40XO, Olympus Life Science, Waltham, MA, USA) were 50 mm, 300 mm, and 5 mm, respectively. The term enclosed in the second brace is for converting the mechanical angle in the scanning mirrors to the driving voltage. *C* is a constant corresponding to the subscript, and the values for the galvanometer mirror and the objective lens are 0.25 and 2.2287, respectively. For the axial scan, the Z positioner unit moves with the objective lens, resulting in shifting the focal spot axially. As a Z positioner, a piezo objective positioner (MIPOS-250, Piezosystem Jena, Jena, Germany) is driven with a piezo controller (NV 40/1 CLE, Piezosystem Jena), and its driving voltage to the axial position is set as shown in Equation (3):

(3) $$V_{Z\left(l\right)}=\left(Z_{0}+\Delta Z\cdot l\right)\times C_{Z}$$

where *V_Z_*_(*l*)_ in mV is a driving voltage of Z positioner, *Z*_0_ in μm is an initial axial position, Δ*Z* in μm is an axial step size, *l* is a layer number to be scanned, and *C_Z_* in mV/μm is a constant that converts the axial position value into the driving voltage. The constant is set to 0.05, taking into account the movement of 1 μm per 50 mV. In the proposed IoT-TPLSM, the 3D scanner module controlled both the laser scanner unit and Z positioner unit, which consisted of an MCU and DAC instead of PC-based DAQ system. The 3D imaging information, such as the image size in pixel numbers, the imaging area in μm, number of axial layers, and axial step size in μm, was transmitted to the MCU from the remote users via IoT, and the MCU generated the waveforms of sawtooth with different periods for three-axis scanning, based on this information. In the 3D scanner module, driving voltages calculated from Equations (2) and (3) were converted via a 16-bit DAC (AD5764R, Analog Devices, Wilmington, MA, USA), as seen in Figure A2a, and these were delivered to the laser scanner and the Z positioner, respectively, to operate IoT-TPLSM remotely.

#### 2.1.3. Photon Counter Module

**Figure 2:**
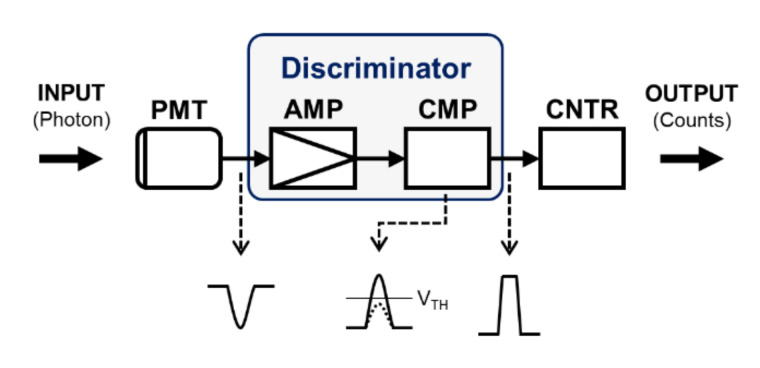
Configuration of the photon counter module. PMT: photomultiplier tube; AMP: amplifier; CMP: comparator; CNTR: counter.

In traditional TPLSM, as shown in Figure 2, the fluorescence signal emitted from the sample is detected using a photomultiplier tube (PMT, H10682-01, Hamamatsu Photonics, Hamamatsu, Japan). As a single photon is transformed to an electron and it is multiplied in the PMT, a series of current pulses is generated. In the amplifier, it is converted and amplified to a series of voltage pulses. Only pulses above the preset threshold voltage level (V_TH_) are altered to digital pulses in the comparator. The intensity of the fluorescence signal can be quantified by counting them through the DAQ system and it is stored in PC. However, In the IoT-TPLSM, the photon counter module quantitated the number of photons detected with a customized photon discriminator and a pulse counter (LS7366R, LSI computer systems, Melville, NY, USA), and it was transferred to remote users via IoT to construct 3D image stacks in the web service.

Besides, a single image was created based on the number of photons detected during pixel residence time at each pixel by raster scan in the laser scanner unit, and the 3D scanner module should be synchronized with the photon counter module to do it. Therefore, handshaking was established by transmitting a 2-bit flag wired into two digital input/output pins in each MCU.

### 2.2. Software Design

**Figure 3:**
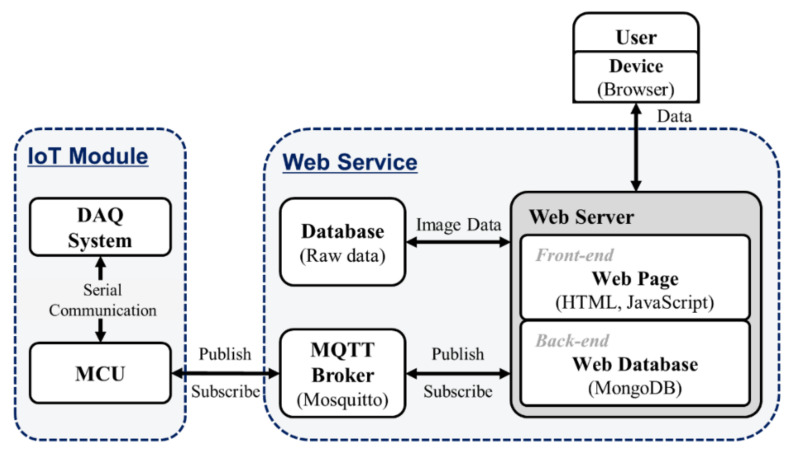
Software configuration of the IoT-TPLSM.

The software configuration for IoT-TPLSM is shown in Figure 3. It is divided into two main parts: IoT module programming and web service programming. The MCU in each IoT module has a serial communication program that enables data exchange between the MCU and DAQ system and MQTT client program that enables the communication between the IoT module and web service with MQTT protocol. A web service functions users to send commands to the IoT actuator modules and receive data from the IoT sensor modules via web browsers in the terminal or PC. It includes an MQTT broker, web server, and database. The MQTT broker connects the web server and MQTT clients, the web server operates the web pages, and the database stores the information for the web pages and the raw data from the DAQ system.

#### 2.2.1. Serial Communication

In this study, an MCU was programmed in the Arduino integrated development environment, and data were transmitted and received by the main device of each module through serial communication. In the laser power controller module, ADC transmitted the voltage readout for the laser power to the MCU through the inter-integrated circuit (I2C) communication, and the MCU delivered the position command as a feedback control signal to the rotary motor via the universal asynchronous receiver/transmitter (UART) RS232 communication. In the 3D scanner module, position command was given to DAC using the serial peripheral interface (SPI) communication. In the photon counter module, the number of photons was passed to the MCU through SPI communication.

#### 2.2.2. MQTT Broker

The MQTT back-end had a data transfer through the Mosquitto broker, specifying the host address and the port to be accessed by the server and MCUs. Datasets were transmitted (publish) and received (subscribe) in the JavaScript object notation (JSON) format on separate channels (topics) for the function of each module and server. The JSON format was used in various programming languages and platforms, and it was easy to exchange data between different systems through parsing [20]. Table 1 shows examples of the dataset formats used in this study.

The MQTT protocol provides three levels for quality of service (QoS) to ensure communication stability: from Level 0, which does not guarantee the QoS, to Level 2, which guarantees the highest QoS [21]. However, Level 2 has a disadvantage in speed performance since it tracks the handshaking process of the messages. In this study, QoS Level 1 was selected, and stability was guaranteed by including page and line information in the dataset.

#### 2.2.3. Web Service

**Figure 4:**
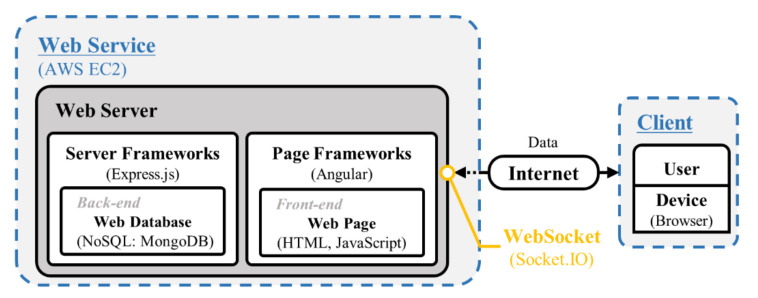
Configuration of the web service.

Figure 4 shows a detailed configuration of the web service. In this study, Amazon web service (AWS EC2, Amazon Web Services, Inc., Seattle, WA, USA) was selected as a web service, which is operated on the Ubuntu OS. The web server performs server-side scripting with the code written using JavaScript and was built through the Node.js-based Express web framework. Express is extensible, so there is no unnecessary interference in writing code and can be easily extended to third-party libraries, and an application programming interface (API) can be created or called quickly and easily through hypertext transfer protocol (HTTP) utility methods and middleware. WebSocket is a hypertext markup language 5 (HTML5) protocol that forms a dynamic two-way connection channel between a user’s browser and a server. It is possible to send a message to the server through the WebSocket API and receive a response without a request. However, HTML5 may not be supported by older browsers. In consideration of compatibility issues between the browsers or with previous versions, the cross-platform WebSocket API, Socket.IO, was used to transmit data messages from the web server. At the front-end of the web server, HTML and JavaScript pages were constructed through the Angular framework. The Angular framework, which is Google’s open-source JavaScript framework for single-page application (SPA) development, has most of the functions required for front-end development of not only web applications but also mobile environments and desktop applications.

In the back-end, a general information database was created using MongoDB, which is a Not Only SQL (NoSQL) database. MongoDB can process most queries quickly with its powerful indexing function, and its processing time is faster than that of MySQL in terms of read and write [22]. Since all data is stored in JSON format, it is very easy to use with MQTT, which transmits and receives data. In addition, The MQTT connection was restricted by receiving user information and reservation information. This was to implement a minimal security system at the web server level because MQTT does not have a separate security system. Finally, result images were saved as data files and could be viewed through the server.

## 3. Results

### 3.1. Functional Validation at the Module Level

**Figure 5:**
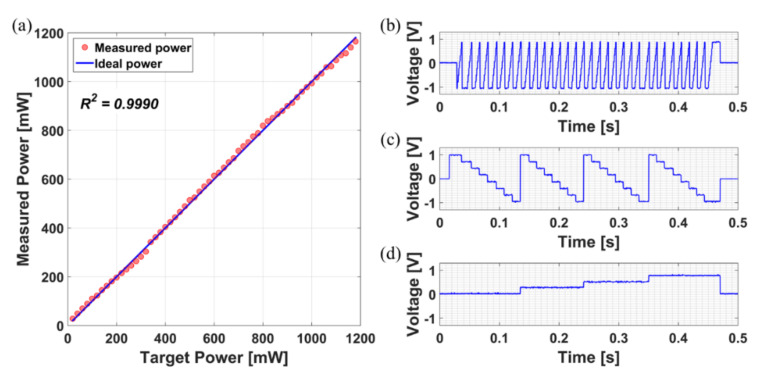
Verification graphs of (**a**) the laser power controller and the 3D scanner for the (**b**) X−axis, (**c**) Y−axis, and (**d**) Z−axis.

Figure 5 shows the results of the independent online operation for each module constituting the hardware. Here, modules were operated using the extension program “MQTTBox” to directly transfer the JSON-formatted datasets. The laser power controller module was controlled through a web browser and the results were validated using a calibrated laser power meter (PowerMax PM10, Coherent). The laser optical power in Figure 5a was measured from 20 mW to 1200 mW in 20 mW steps. The coefficient of determination in the linear regression, i.e., the R^2^, was 0.9990, showing a high correlation. Although the measurement is performed according to Equation (1), the value is saturated when the photocurrent exceeds a certain level. Therefore, the appropriate range should be set by adjusting the resistance value. In this study, we focused on 3D optical microscopy and set the range to low power to expand into biopsy or in-vivo studies. To ensure stability, the input outside the range was processed to output the target value as the default value of 100 mW.

Synchronized 3D scanning was performed using units of the laser scanner and the Z positioner. It was run briefly only for functional verification of remote operation. The FOV of 50 μm × 50 μm was set to 8 × 8 pixels, imaging speed per pixel was set to 10 μs, and depth was set to 4 layers in 5 μm increments. The synchronized drive following Equation (2) was confirmed as shown in Figure 5b–d.

### 3.2. Performance Comparison for IoT-TPLSM at the System Level

#### 3.2.1. Web Service for IoT-TPLSM

**Figure 6:**
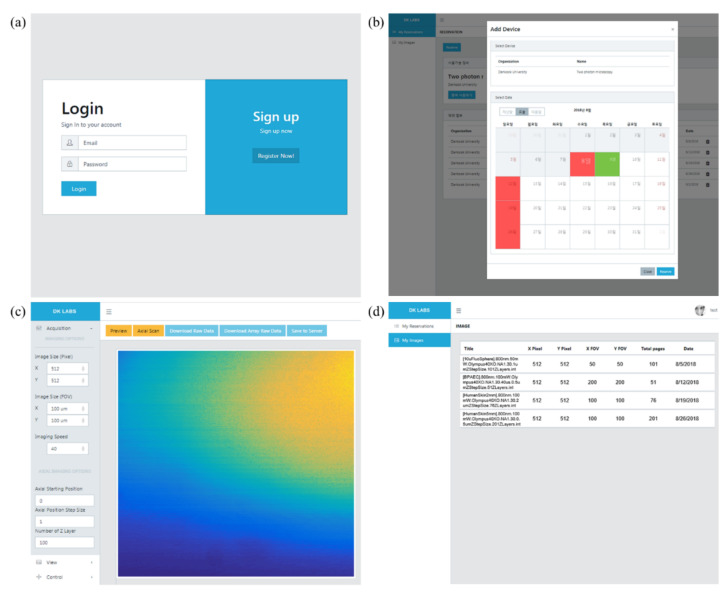
Page screenshots of the web service user interface: (**a**) login and sign up page; (**b**) equipment selection page; (**c**) equipment operation page; and (**d**) data log page.

In order to operate IoT-TPLSM remotely, the users need to access a web service for microscopes via a web browser, and the procedure for imaging biological samples is demonstrated as follows. The users are supposed to login first through the login and signup page shown in Figure 6a. Then, users choose which microscope they use and reserve which dates they will image a sample with the selected microscope on the web page that appears in Figure 6b. On the date when the microscope is reserved, they image a sample by controlling the microscope remotely on the page presented in Figure 6c, which provides functions such as setting parameters, monitoring images, and saving the 3D image stack. After imaging, all the information for the 3D image stack log is displayed, as shown in Figure 6d. The 3D image stacks stored in the cloud service can be retrieved later. The detailed descriptions about user interface panels for the web service are in Appendix B. Such a whole remote imaging procedure was confirmed by acquiring the following images step-by-step.

#### 3.2.2. Precision Comparison with Fluorescent Microsphere Imaging

**Figure 7:**
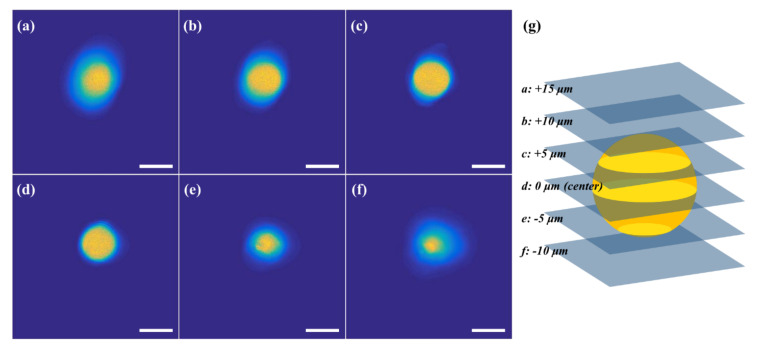
The images for a fluorescent microsphere at different depths from the center: (**a**) +15 μm; (**b**) +10 μm; (**c**) +5 μm; (**d**) 0 μm; (**e**) −5 μm; and (**f**) −10 μm. (scale bar: 10 μm). (**g**) Illustration of a 3D image of a fluorescent microsphere.

As a standard for 3D fluorescence imaging, yellow-green fluorescent microspheres (F8836, Molecular Probes, Eugene, OR, USA) with a nominal diameter of 10 μm were imaged to evaluate the image pixel precision. The 3D image stack was obtained up to a depth of 100 μm with 1 μm steps by setting the laser power to 50 mW at a wavelength of 800 nm and FOV of 50 μm × 50 μm, which corresponds to 512 × 512 pixels. Some images extracted with a 5 μm step are presented in Figure 7a–f.

**Figure 8:**
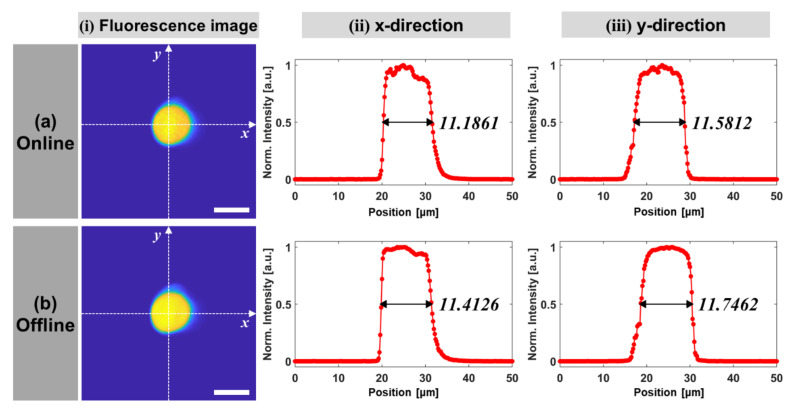
Quantitative comparison of the performance of (**a**) online and (**b**) offline systems. (**i**) Fluorescent microsphere images, and the corresponding diameter of the (**ii**) x-direction and (**iii**) y-direction intensity profiles (scale bar: 10 μm).

Images extracted in the layer for the microsphere center in the axial direction are shown in Figure 8i for quantitative comparison between images acquired online and offline. The normalized intensity profiles along the x and y direction passing through the center of a microsphere are plotted in Figure 8ii,iii, respectively. The microsphere’s diameter was expressed in terms of full width at half maximum (FWHM) of the intensity profile after applying the piecewise cubic Hermite interpolation. In the IoT-TPLSM, its diameters were measured as 11.1861 μm and 11.5812 μm on the x and y direction, respectively. In the conventional TPLSM as a control, they were measured as 11.4126 μm and 11.7462 μm on the x and y direction, respectively. The error on the x-direction was 1.98%, and that on the y direction was 1.40%, which confirmed that similar precision was maintained between the online (IoT-TPLSM) and offline (TPLSM) imaging results.

### 3.3. Demonstration of IoT-TPLSM

#### 3.3.1. 3D Fluorescence Imaging at the Cellular Level

**Figure 9:**
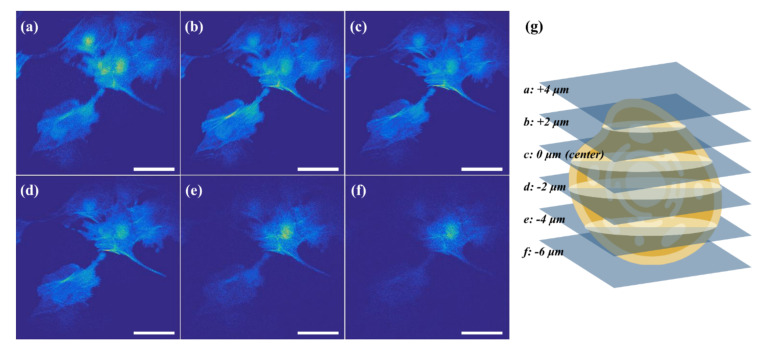
The images for the stained bovine pulmonary artery endothelial (BPAE) cells at different depths from the center: (**a**) +4 μm; (**b**) +2 μm; (**c**) 0 μm; (**d**) −2 μm; (**e**) −4 μm; and (**f**) −6 μm (scale bar: 10 μm). (**g**) Illustration of the 3D image of BPAE cells.

As an application of a biological sample, a 3D image stack for the stained bovine pulmonary artery endothelial (BPAE) cells (F36924, Molecular Probes), was obtained up to a depth of 25 μm, with 0.5 μm steps with the laser power at 100 mW at a wavelength of 800 nm and FOV of 200 μm × 200 μm, which corresponds to 512 × 512 pixels. Some images extracted with 2 μm steps are displayed in Figure 9a–f. Although BPAE cells were stained using three fluorescent dyes, only F-actin stained with Alexa Fluor 488 phalloidin and nuclei stained with DAPI were clearly identified. Mitochondria stained with MitoTracker Red CMXRos was not detected because the laser wavelength is out of range on its excitation wavelengths. Operating the IoT-TPLSM remotely, the 3D fluorescent image at the cellular level was obtained with high similarity to the offline system.

#### 3.3.2. 3D Autofluorescence Imaging at the Tissue Level

**Figure 10:**
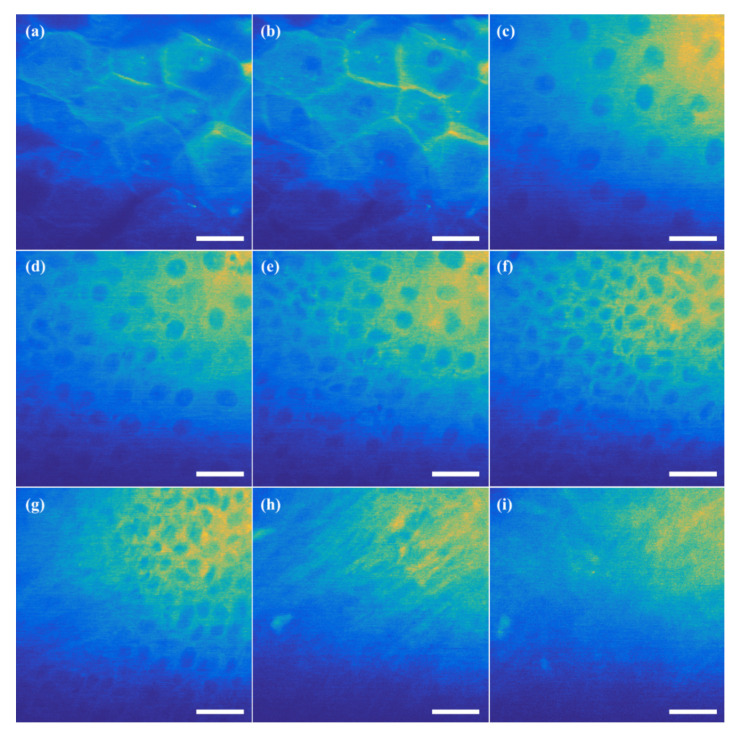
The images of the human skin tissue at different depths from the surface: (**a**) stratum corneum without nuclei at 17 μm, (**b**) epidermal cells with nuclei 17 μm, (**c**) granular layer at 45 μm, (**d**) cell membranes at 54 μm, (**e**) stratum spinosum at 60 μm, (**f**) cubic basal cells at 66 μm, (**g**) dermal fibrous tissue at 76 μm, (**h**) collagenous fiber tissue at 82 μm, and (**i**) amorphous collagen tissue at 96 μm (scale bar: 20 μm).

Ex-vivo human skin tissue provided from Dankook University Hospital was also imaged as a 3D autofluorescence image stack, which is expressed in the unstained tissue. The 3D images were acquired up to a depth of 100 μm with a 2 μm step by setting the laser power of 100 mW at a wavelength of 800 nm and FOV of 100 μm × 100 μm corresponds to 512 × 512 pixels. Some images extracted at different depths are represented in Figure 10a–i. Starting from the stratum corneum without nuclei on the surface of the skin, the keratinocyte in the epidermis, the dermal-epidermal junction, and the collagenous fiber tissue in the dermis were definitely recognized. It was observed that the stratum corneum without nuclei existed at 17 μm; nuclei in the epidermal cells began to appear at 22 μm. It was noticed that the keratinocyte nuclei were distributed as the granular layer at 45 μm, cell membranes were maintained at 54 μm, polygonal keratinocytes as the stratum spinosum at 60 μm, cubic basal cells at 66 μm, and the dermal–epidermal junction where the dermal fibrous tissue and some cells were mixed at 76 μm. It was also found that the collagenous fiber tissue and the amorphous collagen tissue in the dermis were located at 82 μm and 96 μm, respectively. It was shown that the 3D image of the label-free ex-vivo human skin tissue was obtained successfully by operating IoT-TPLSM remotely.

## 4. Discussion and Conclusions

In this study, an IoT-based remote control system for shared research equipment was proposed and implemented. The offline system for equipment was expanded to the IoT convergence platform and cloud, resulting in transforming the online system. Moreover, a single synchronized system with independently configured MCUs and the web service interface for a customized DAQ were completed. Using the remote full-duplex, it was confirmed that the remote operation for various research equipment can be additionally and alternatively utilized in diverse research fields. It is also expected that the IoT-based research equipment sharing system allows researchers at a remote site to set up an experiment as well as check and save the result at their own will.

By taking IoT-TPLSM as an example application, the stained cellular images and the autofluorescent tissue images were obtained. As a result, it was confirmed that performances for the online system, such as the image acquisition time, the image quality, and GUI for image acquisition, were almost the same as those for the offline one. The image distortion shown under 2% can be easily corrected with the calibration for driving voltages. Besides, as the proposed remote sharing system used the web service and MCU, access for the IoT module was fully granted to the remote users to operate shared equipment freely. Simultaneously, since the shared equipment working with the MQTT protocol via IoT was independent of the computer itself, unexpected OS problems were eliminated, and the operating stability was more secured.

The remote operation of research equipment was executed through a wireless network. However, various attempts to overcome its vulnerability are needed, since wireless networks are relatively insecure compared to wired networks. As the MQTT protocol supports QoS, the optimal QoS for real-time communication can be set [23]. In addition, while using the MQTT, a standby database can be placed between the gateway and the server [24], and the current protocol can be upgraded or attached parallel to other wireless communication protocols to address network failures [25,26,27]. By applying such stabilization to the system in this study, it is believed it would ensure the rapid and stable remote operation of shared equipment, even using a wireless network.

The IoT-based remote sharing system is expected to provide a realistic solution for equipment utilization and thus can be used as a basic technology in many industries. In the manufacturing field, it can be applied to a smart factory or for hybrid manufacturing implemented with remote robot systems [28]. In the biomedical field, the remote robot system could enable automatic sample replacement and remote experiments in a single queue, and the remote operation system can be extended to telemedicine with deep learning to aid in disease diagnosis in the clinic [29,30,31]. The proposed remote sharing system is also expected to serve as a window for network formation and integration between researchers in various fields through the remote sharing of various research equipment and to open a new chapter in research and development areas.

## Figures and Tables

**Table 1:** Examples of a message set in JSON format.

**Topic**	*{device ID}/mirror/cmd*	*{device ID}/mirror/data*
**Clients**	Publish client: Server Subscribe client: Device	Publish client: Device Subscribe client: Server
**JSON format dataset**	{ “command”: “axialscan”, “options”: { “xPixel”: number, “yPixel”: number, “xFov”: number, “yFov”: number, “imagingSpeed”: number, “ch1”: boolean, “ch2”: boolean, “ch3”: boolean, “axialStepSize”: number, “axialZero”: number, “numOfZLayer”: number } }	{ “message”: “axialdata”, “data”: { “channel”: string, “totalPages”: number, “page”: number, “xPixel”: number, “yPixel”: number, “line”: number, “imagingData”: Uint16Array } }

**Figure A3:**
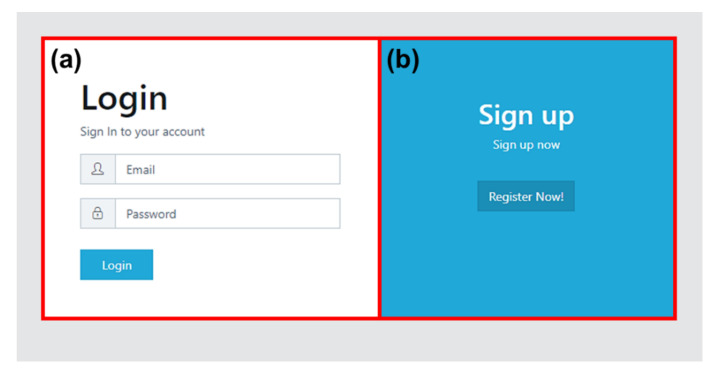
The user interface of the login and sign up page: (**a**) login panel and (**b**) sign-up panel.

**Figure A4:**
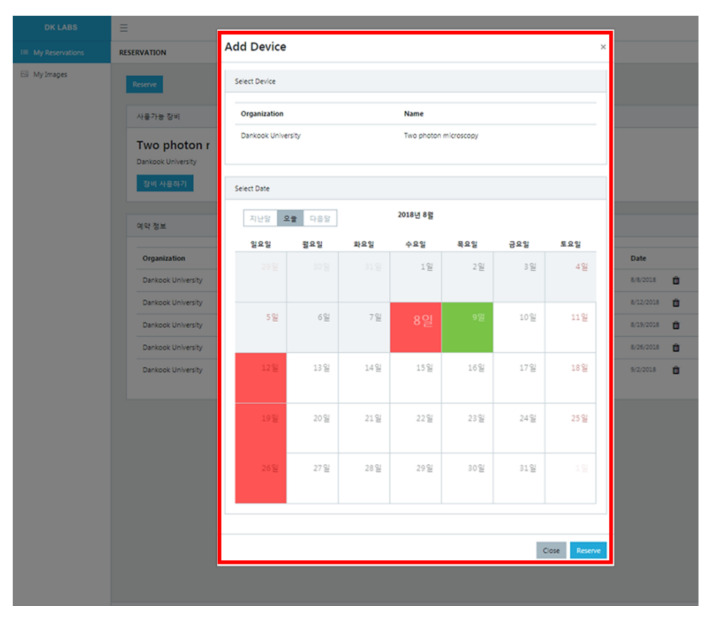
The user interface of the equipment selection page. The red box is a calendar for reservations.

**Figure A5:**
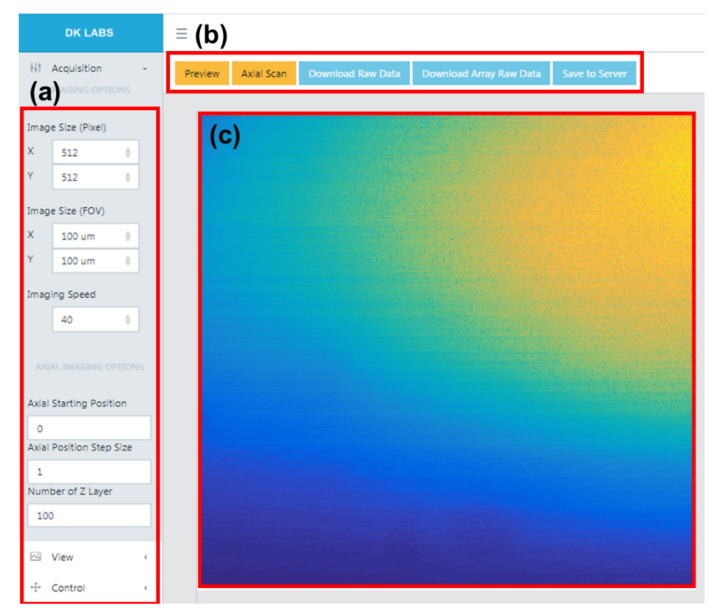
The user interface of the equipment operation page: (**a**) parameter-setting panel; (**b**) operation button panel; and (**c**) result image panel.

**Figure A6:**
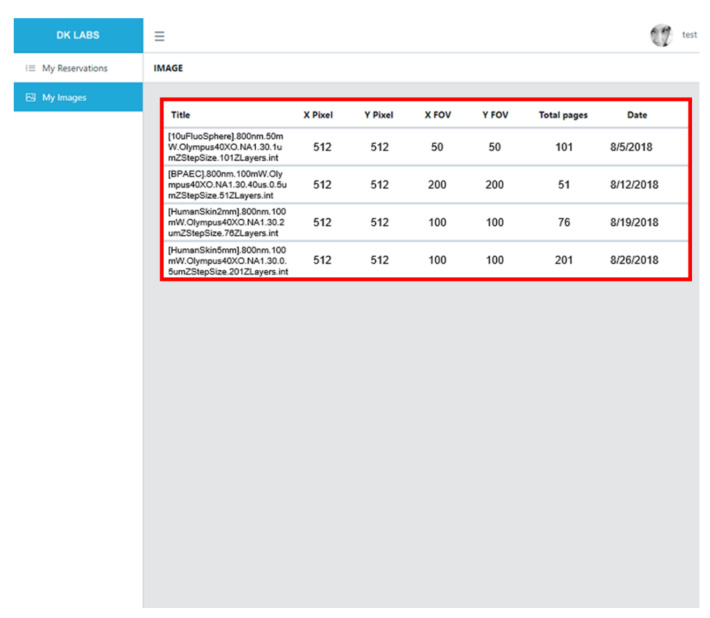
The user interface of the data log page. The red box is a result data log panel of shared equipment.

## Data Availability

The hardware data presented in this study are available in the article. The software data presented in this study are available on request from the corresponding author. Source codes are not publicly available due to the security policy of the funding organization.
